# Characteristics and clinical outcomes in young‐onset cholangiocarcinoma

**DOI:** 10.1002/cam4.6063

**Published:** 2023-05-22

**Authors:** Sarah Reddy, Suleyman Yasin Goksu, Nina N. Sanford, Radhika Kainthla, David Hsiehchen, Aravind Sanjeevaiah, Amy L. Jones, Georgios Karagkounis, Salwan Al Mutar, Chul Ahn, Muhammad S. Beg, Syed M. Kazmi

**Affiliations:** ^1^ Department of Internal Medicine UT Southwestern Medical Center Dallas Texas USA; ^2^ Division of Hematology and Oncology UT Southwestern Medical Center Dallas Texas USA; ^3^ Department of Radiation Oncology UT Southwestern Medical Center Dallas Texas USA; ^4^ Division of Surgical Oncology UT Southwestern Medical Center Dallas Texas USA; ^5^ Department of Population and Data Sciences UT Southwestern Medical Center Dallas Texas USA; ^6^ Science 37 Durham North Carolina USA

**Keywords:** age of onset, cholangiocarcinoma, hepatobiliary neoplasms, treatment outcome

## Abstract

**Background:**

While the incidence of cholangiocarcinoma is rising, little is known about young‐onset disease. We compared clinical characteristics and outcomes between patients with young‐onset cholangiocarcinoma, diagnosed between the ages of 18 and <50 years, and patients with typical‐onset cholangiocarcinoma, diagnosed at age 50 years or greater.

**Methods:**

We used the National Cancer Database to identify patients with young‐onset cholangiocarcinoma (n = 2520) and typical‐onset cholangiocarcinoma (*n* = 23,826). We compared the frequency of demographic and clinical characteristics between the two groups. We compared overall survival between the two groups using multivariable Cox regression analysis after adjusting for age, gender, race/ethnicity, comorbidity, facility type, tumor location, tumor stage, surgical status, and receipt of radiotherapy, chemotherapy and surgery.

**Results:**

When compared to patients with typical‐onset disease (median age 68 years), patients with young‐onset cholangiocarcinoma (median age 44 years) were more likely to be non‐White (35.0% vs. 27.4%, *p* < 0.01), and had lower overall comorbidity burden. Patients with young‐onset disease had a greater proportion of intrahepatic cholangiocarcinoma (56.0% vs. 45.5%, *p* < 0.001) and stage IV disease (50.5% vs. 43.5%, *p* < 0.001). Younger patients were more likely than typical‐onset patients to receive definitive surgery (30.9% vs. 25.0%, *p* < 0.001), radiation (27.7% vs. 19.6%, *p* < 0.001) and chemotherapy (73.1% vs. 50.1%, *p* < 0.001). In adjusted analyses, patients with young‐onset disease had a 15% decreased risk of death, compared with patients with typical‐onset disease (HR 0.85 [95% CI 0.80–0.89], *p* < 0.001).

**Conclusions:**

Patients with young‐onset cholangiocarcinoma may represent a demographically and clinically distinct group from those with more typical‐onset disease.

## Graphical abstract

Young‐onset cholangiocarcinoma is associated with distinct demographic and clinical characteristics. Patients with young‐onset cholangiocarcinoma have a 15% decreased risk of death compared to patients with typical‐onset disease after adjusting for demographics, comorbidity, and treatment.

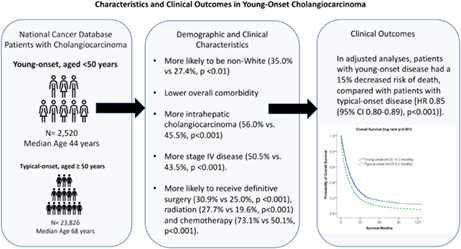

## 1 INTRODUCTION

The incidence of young‐onset gastrointestinal malignancy is rising.^1^, ^2^ Younger patients with gastrointestinal malignancy appear to have distinct demographic characteristics, disease presentation, histopathologic features, and clinical outcomes, when compared to patients with typical‐onset disease.^1^, ^2^, ^3^ This phenomenon has been most studied for colorectal cancer, the prototypic solid tumor malignancy with rising incidence among patients under the age of 50 years.^2^

Cholangiocarcinoma is a rare but highly fatal malignancy that arises in the biliary tree.^4^ The incidence of cholangiocarcinoma, especially intrahepatic cholangiocarcinoma, continues to rise in the United States.^5^ One United States population‐based study estimates that the annual percent rise in the incidence of cholangiocarcinoma between 2003 and 2012 was between 0.16% and 4.36% per year.^5^ The median age of diagnosis of cholangiocarcinoma in the United States is between 67 and 72 years of age.^5^ Younger patients aged <50 years with cholangiocarcinoma represent an important but relatively understudied group. Little is known about the incidence and demographic characteristics of cholangiocarcinoma among these young patients. There is conflicting data on mortality in these young patients, with one analysis finding improved outcomes,^6^ while another analysis suggests worse mortality in young patients with cholangiocarcinoma.^7^

A recent analysis of patients aged 15 through 45 years with cholangiocarcinoma demonstrated that younger patients were more likely to carry additional sex combos like 1 (ASXL1) and lysine methyltransferase 2c (KMT2C) mutations compared with older patients with cholangiocarcinoma.^7^ Despite these potential genetic differences, little is known about clinical outcomes in patients with young‐onset disease. The purpose of this investigation is to describe the demographic and clinical characteristics of patients with young‐onset cholangiocarcinoma in the United States. We also aimed to compare clinical outcomes among patients with young‐onset cholangiocarcinoma and patients with typical‐onset disease and identify subgroups with improved outcomes.

## 2 METHODS

### 2.1 Patient population

This was a retrospective analysis using the National Cancer Database (NCDB), a nationwide, multi‐facility clinical database in the United States sponsored by the American College of Surgeons and the American Cancer Society.^8^ The database contains patient‐level information, including demographics, tumor characteristics, treatment, and clinical outcomes. At present, the NCDB captures 70% of newly diagnosed cancers in the United States and contains more than 34 million historical cancer cases.

**FIGURE 1:**
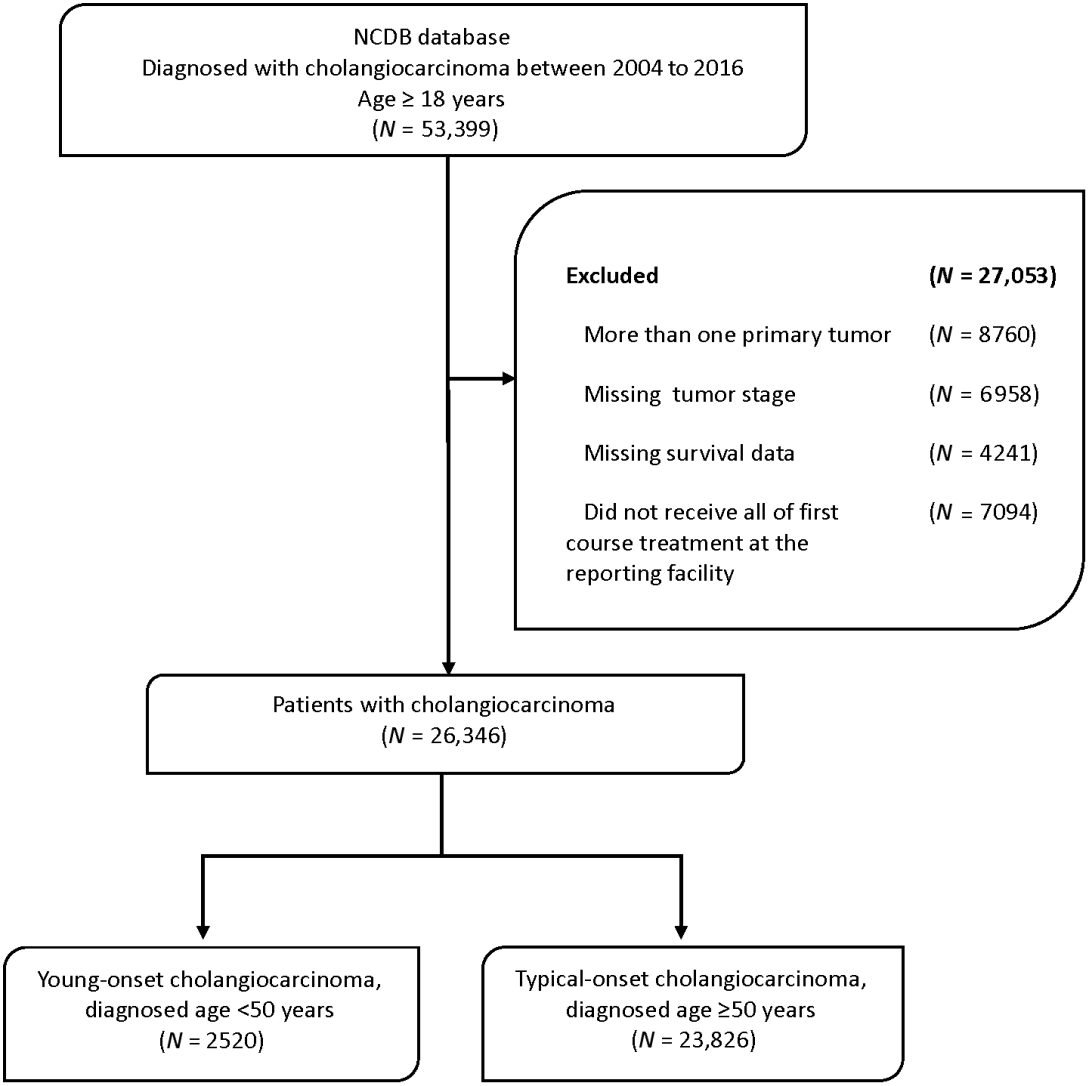
Flow chart of study population section.

The study population included patients aged 18 years or older diagnosed between 2004 and 2016 (Figure 1). Patients with cholangiocarcinoma were identified using International Classification of Diseases 10 (ICD‐10) codes C22.1 or C24.0, and International Classification of Diseases for Oncology 3 (ICD‐O‐3) histology codes 8140 and 8160.^9^, ^10^, ^11^ Young‐onset cholangiocarcinoma patients were diagnosed at age <50 years.^12^ Typical‐onset cholangiocarcinoma was defined as diagnosis at age ≥50 years. We excluded patients who had unknown survival or staging data, more than one primary tumor, or who did not receive their entire first course of treatment at the reporting facility. Patients with missing data were excluded from the regression analyses.

### 2.2 Covariables

We included the following demographic variables: sex, race/ethnicity, year of diagnosis, insurance status, median income quartiles, patient residence (metropolitan vs. non‐metropolitan), travel distance to treatment facility, and Charlson‐Deyo comorbidity index score (0,1, ≥2). The Charlson comorbidity index is a validated measure of overall comorbidity burden composed of 17 disease categories.^13^ Tumor characteristics included stage at diagnosis, nodal involvement (yes/no), presence of lymphovascular invasion (yes/no), and primary location (intrahepatic vs. extrahepatic). The NCDB analytic stage preferentially uses the TNM American Joint Committee on Cancer (AJCC) pathological stage group when available, and defaults to the TNM AJCC clinical stage group if not.^8^ Primary tumor location was categorized as either intrahepatic (ICD‐10 code C22.1) or extrahepatic (ICD‐10 code C24.0). Treatment variables included surgery status, receipt of radiotherapy as part of the initial treatment course, receipt of chemotherapy as part of the initial treatment course, and facility type (academic vs. non‐academic). Definitive surgery was defined using surgery codes of the primary site. For intrahepatic cholangiocarcinoma, these included wedge resection, lobectomy, extended lobectomy, hepatectomy, and bile duct excision. For extrahepatic cholangiocarcinoma, these included simple/partial surgical removal of primary site, total surgical removal of the primary site, and radical surgery.^14^, ^15^ The NCDB participant user file data dictionary describes the acquisition, definitions, and standardization of these variables and can be accessed at https://www.facs.org/quality‐programs/cancer/ncdb/puf.

### 2.3 Statistical analysis

Baseline covariates were summarized using frequencies and percentages. We used the chi‐square test to compare demographic and clinical characteristics between patients with young‐onset cholangiocarcinoma and typical‐onset cholangiocarcinoma. Survival data were extracted from the NCDB vital status variable, which codes participants as either dead or alive at the date of the last contact. Patients who were alive at the last follow‐up were censored. Overall survival was estimated using the Kaplan–Meier method with log‐rank tests. We used multivariate Cox regression analysis to determine whether young‐onset cholangiocarcinoma was significantly associated with overall survival after controlling the effects of gender, race/ethnicity, comorbidity score, facility type, primary location, receipt of radiotherapy, receipt of chemotherapy, and receipt of surgery. Data were presented using hazard ratios (HR) with a 95% confidence interval (CI).

We also performed subgroup analyses for patients with stage I, II, III, and IV disease at diagnosis, and for patients who received definitive surgery, patients who had intrahepatic cholangiocarcinoma, and patients who had extrahepatic cholangiocarcinoma. We used the log‐rank test to compare overall survival in young‐onset cholangiocarcinoma versus typical‐onset cholangiocarcinoma in each of these seven subgroups. Statistical significance was assessed using a two‐sided significance level of 0.05. Analyses were performed using SPSS version 25.0.

## 3 RESULTS

We identified 2520 patients with young‐onset cholangiocarcinoma (median age 44 years) and 23,826 patients with typical‐onset cholangiocarcinoma (median age 68 years). Demographic and clinical characteristics are summarized in Table 1. Patients with young‐onset cholangiocarcinoma were more likely to be male, non‐White, have lower overall comorbidity, and have private insurance compared to patients with typical‐onset cholangiocarcinoma. Patients with young‐onset disease were more likely to have intrahepatic cholangiocarcinoma (56% vs. typical‐onset cholangiocarcinoma 46%, *p* < 0.001). A higher proportion of patients with young‐onset cholangiocarcinoma were diagnosed with stage IV disease (51% vs. typical‐onset cholangiocarcinoma 44%, *p* < 0.001). While there was no significant difference in nodal involvement between the two age groups, patients with young‐onset cholangiocarcinoma were more likely to have tumors that were positive for lymphovascular invasion (45% vs. typical‐onset cholangiocarcinoma 38%, *p* = 0.001). Patients with young‐onset cholangiocarcinoma were more likely to undergo definitive surgery (31% vs. typical‐onset cholangiocarcinoma 25%, *p* < 0.001), receive radiotherapy (28% vs. typical‐onset cholangiocarcinoma 20%, *p* < 0.001), and chemotherapy (73% vs. typical‐onset cholangiocarcinoma 50%, *p* < 0.001), when compared with patients with typical‐onset cholangiocarcinoma. Younger patients were also more likely to undergo treatment at an academic center (64% vs. typical‐onset cholangiocarcinoma 53%, *p* < 0.001).

**TABLE 1:** Baseline demographic and clinical characteristics of patients with young‐onset cholangiocarcinoma and typical‐onset cholangiocarcinoma.

Characteristics	Yong‐onset cholangiocarcinoma	Typical‐onset cholangiocarcinoma	*p*‐value
	*N* = 2520	*N* = 23,826	
Gender			0.008
Male	1373 (54.5)	12,322 (51.7)	
Female	1147 (45.5)	11,504 (48.3)	
Race/ethnicity			<0.001
Non‐Hispanic White	1638 (65.0)	17,292 (72.6)	
Non‐Hispanic Black	281 (11.2)	1986 (8.3)	
Hispanic	296 (11.7)	1867 (7.8)	
Other	305 (12.1)	2681 (11.3)	
Charlson comorbidity score			<0.001
0	2110 (83.7)	16,123 (67.7)	
1	275 (10.9)	5301 (22.2)	
2+	135 (5.4)	2402 (10.1)	
Facility type			<0.001
Academic	1192 (63.7)	12,702 (53.3)	
Non‐academic	680 (36.3)	11,124 (46.7)	
Insurance status			<0.001
Uninsured	194 (8.0)	746 (3.2)	
Private	1636 (67.6)	7597 (32.6)	
Medicaid	423 (17.5)	1357 (5.8)	
Medicare	131 (5.4)	13,272 (57.0)	
Other government	35 (1.4)	297 (1.3)	
Income (USD)			0.08
<38,000	458 (18.5)	4333 (18.5)	
38,000–47,999	547 (22.1)	5090 (21.7)	
48,000–62,999	535 (21.6)	5588 (23.8)	
>63,000	934 (37.8)	8448 (36.0)	
Rurality			0.26
Metropolitan	2071 (85.0)	19,489 (84.1)	
Non‐metropolitan	365 (15.0)	3672 (15.9)	
Travel distance to treatment facility			<0.001
<12.5 miles	1020 (40.6)	11,386 (48.0)	
12.5–49.9 miles	826 (32.8)	7557 (31.8)	
≥50 miles	669 (26.6)	4800 (20.2)	
Primary location			<0.001
Intrahepatic	1410 (56.0)	10,834 (45.5)	
Extrahepatic	1110 (44.0)	12,992 (54.5)	
Nodal status			0.08
Negative	436 (47.3)	3655 (50.4)	
Positive	485 (52.7)	3599 (49.6)	
Lymphovascular invasion			0.001
No	289 (54.8)	3061 (62.5)	
Yes	238 (45.2)	1838 (37.5)	
Stage at diagnosis			<0.001
I	342 (13.6)	4297 (18.0)	
II	533 (21.2)	5851 (24.6)	
III	373 (14.8)	3323 (13.9)	
IV	1272 (50.5)	10,355 (43.5)	
Surgery			<0.001
None	1598 (69.1)	16,518 (75.0)	
Definitive	715 (28.3)	5500 (22.7)	
Non‐definitive	65 (2.6)	546 (2.3)	
Radiotherapy			<0.001
No	1816 (72.3)	19,038 (80.4)	
Yes	695 (27.7)	4652 (19.6)	
Chemotherapy			<0.001
No	654 (26.9)	11,429 (49.9)	
Yes	1773 (73.1)	11,488 (50.1)	
Overall survival			<0.001
Median (months)	14.0	9.3	

*Note*: Young‐onset cholangiocarcinoma diagnosed age <50 years, typical‐onset cholangiocarcinoma diagnosed at age ≥50 years.

**FIGURE 2:**
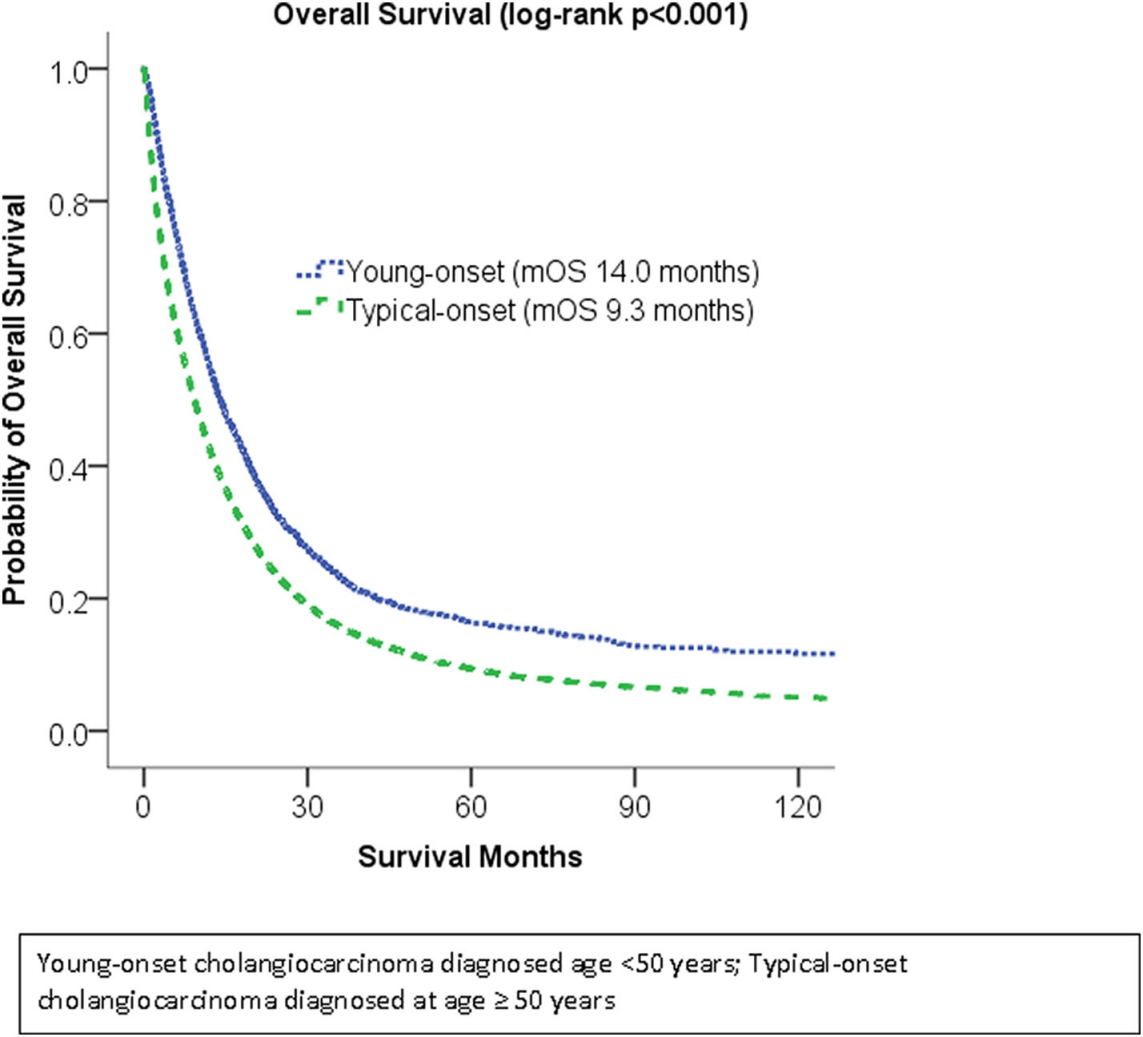
Overall survival among patients with young‐onset cholangiocarcinoma versus typical‐onset cholangiocarcinoma.

Patients with young‐onset cholangiocarcinoma had a median survival of 14.0 months from the time of diagnosis, compared to 9.3 months for typical‐onset cholangiocarcinoma (*p* < 0.001). In unadjusted analysis, young‐onset cholangiocarcinoma was associated with a 28% decreased risk of death compared to patients with typical‐onset cholangiocarcinoma. After adjusting for gender, race/ethnicity, comorbidity score, facility type, primary location, stage, radiotherapy, chemotherapy, and surgery, patients with young‐onset cholangiocarcinoma had a 15% decreased risk of death compared to patients with typical‐onset cholangiocarcinoma (HR 0.85 [95% CI 0.80–0.89], *p* < 0.001) (Table 2). Cumulative survival in the adjusted analysis is presented in Figure 2. Female sex, Hispanic ethnicity, lower comorbidity, extrahepatic disease, early stage at diagnosis, treatment at an academic center, and receipt of radiotherapy, chemotherapy, and surgery were all associated with decreased mortality risk. Median overall survival and hazard ratios for mortality by stage group for patients with young‐onset cholangiocarcinoma and typical‐onset cholangiocarcinoma are presented in Table 3. Among patients with stage I disease, those with young‐onset cholangiocarcinoma had a median overall survival of 48.4 months, compared with 15.3 months for patients with typical‐onset cholangiocarcinoma (*p* < 0.001). The survival difference was present but less pronounced for patients with stage IV disease, in whom young‐onset cholangiocarcinoma was associated with a median survival of 8.6 months versus 5.2 months for typical‐onset cholangiocarcinoma (*p* = 0.013).

**TABLE 2:** Multivariable Cox regression analysis for overall survival in patients with young‐onset cholangiocarcinoma and typical‐onset cholangiocarcinoma.

Characteristics	Overall survival	
	HR (95% CI)	*p*‐value
Age		
Typical‐onset cholangiocarcinoma	Ref	
Young‐onset cholangiocarcinoma	0.85 (0.80–0.89)^a^	<0.001
Gender		
Male	Ref	
Female	0.90 (0.88–0.93)	<0.001
Race/ethnicity		
Non‐Hispanic White	Ref	
Non‐Hispanic Black	1.01 (0.96–1.06)	0.85
Hispanics	0.84 (0.79–0.88)	<0.001
Other	0.95 (0.91–0.99)	0.01
Charlson comorbidity score		
0	Ref	
1	1.13 (1.09–1.17)	<0.001
2+	1.40 (1.33–1.46)	<0.001
Facility type		
Academic	Ref	
Non‐academic	1.25 (1.21–1.28)	<0.001
Primary location		
Intrahepatic	Ref	
Extrahepatic	1.07 (1.04–1.10)	<0.001
Stage		
I	Ref	
II	1.49 (1.42–1.56)	<0.001
III	1.70 (1.62–1.79)	<0.001
IV	2.25 (2.15–2.35)	<0.001
Radiotherapy		
No	Ref	
Yes	0.84 (0.81–0.87)	<0.001
Chemotherapy		
No	Ref	
Yes	0.51 (0.49–0.52)	<0.001
Surgery		
None	Ref	
Definitive	0.35 (0.34–0.36)	<0.001
Non‐definitive	0.45 (0.41–0.50)	<0.001

Abbreviations: CI, confidence interval; HR, hazard ratio.

^a^ Adjusted for gender, race/ethnicity, comorbidity score, facility type, primary location, stage, radiotherapy, chemotherapy, and surgery.

**TABLE 3:** Median overall survival and hazard ratios for mortality based on stage at diagnosis.

	Univariable		Multivariable^a^	
	Median OS (months)	*p*‐value	HR (95% CI)	*p*‐value
Stage I		<0.001		
YOCC	48.4		Ref	
TOCC	15.3		1.86 (1.54–2.25)	<0.001
Stage II		<0.001		
YOCC	23.0		Ref	
TOCC	15.4		1.22 (1.07–1.38)	0.002
Stage III		<0.001		
YOCC	15.6		Ref	
TOCC	11.6		1.12 (0.98–1.29)	0.12
Stage IV		<0.001		
YOCC	8.6		Ref	
TOCC	5.2		1.10 (1.02–1.18)	0.013

Abbreviations: CI, confidence interval; HR, hazard ratio; OS, overall survival; TOCC, typical‐onset cholangiocarcinoma diagnosed at age ≥50 years; YOCC, Young‐onset cholangiocarcinoma diagnosed at age <50 years.

^a^ Adjusted for gender, race/ethnicity, comorbidity score, facility type, primary location, radiotherapy, chemotherapy, and surgery.

**FIGURE 3:**
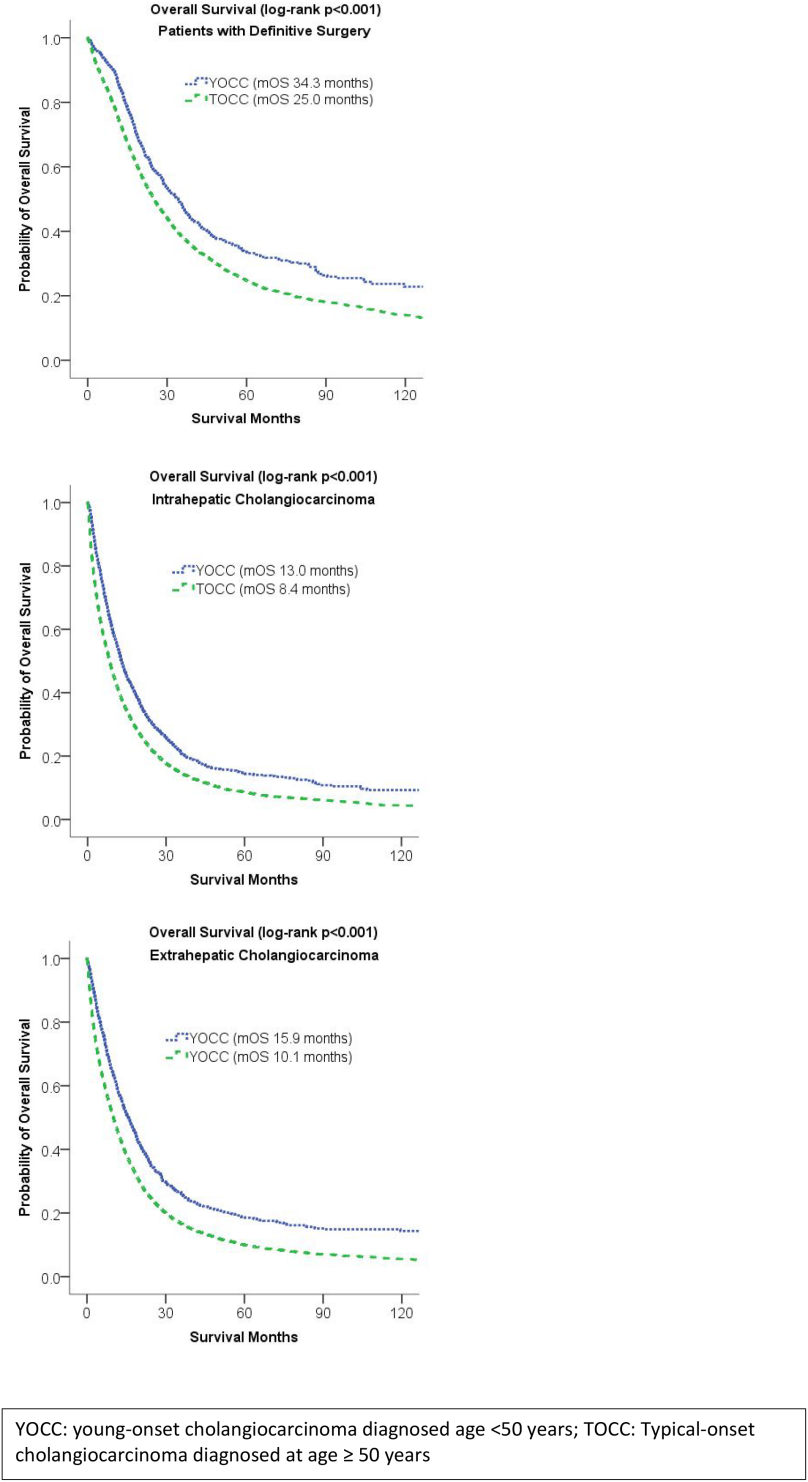
Overall survival among patients who received definitive surgical treatment for cholangiocarcinoma, and among patients with intrahepatic and extrahepatic cholangiocarcinoma.

Among the subgroup of patients who underwent definitive surgery, those with young‐onset cholangiocarcinoma had a longer median survival from the time of diagnosis, compared with patients with typical‐onset cholangiocarcinoma (34.3 vs. 25.0 months, respectively, *p* < 0.001). Among patients with intrahepatic disease, young‐onset cholangiocarcinoma was associated with longer median survival (13.0 months vs. typical‐onset cholangiocarcinoma 8.4 months, *p* < 0.001). This survival benefit was similar in the subgroup of patients with extrahepatic disease (median survival young‐onset cholangiocarcinoma 15.9 months vs. typical‐onset cholangiocarcinoma 10.1 months, *p* < 0.001). Cumulative survival for subgroups undergoing surgery and for subgroups with intrahepatic cholangiocarcinoma and extrahepatic cholangiocarcinoma are presented in Figure 3.

## 4 DISCUSSION

In this analysis, we found that patients with young‐onset cholangiocarcinoma have distinct demographic and clinical characteristics compared with patients with typical‐onset cholangiocarcinoma. Despite having a greater proportion of stage IV disease, patients with young‐onset cholangiocarcinoma were more likely to receive chemotherapy, radiation, and definitive surgery when compared with older patients. We observed a 15% decreased risk of overall mortality among patients with young‐onset cholangiocarcinoma compared to patients with typical‐onset cholangiocarcinoma. This survival benefit was independent of comorbidity burden, tumor stage, and receipt of radiotherapy, chemotherapy, and surgery. The survival benefit in young‐onset cholangiocarcinoma was most notable for early stage disease but was also present among patients diagnosed with stage IV disease. Among patients who received definitive surgery, those with young‐onset disease had a significantly prolonged median overall survival than those with typical‐onset disease.

To our knowledge, only one other study has directly compared patients with young‐onset cholangiocarcinoma to those with typical‐onset disease. This study included 26 patients with young‐onset disease and may not have been powered to detect mortality differences. The analysis found no significant mortality difference between adolescents and young adults with cholangiocarcinoma (aged 15 through 45 years) compared with older adults with cholangiocarcinoma.^7^ Our findings are consistent with a separate US population‐based analysis that finds that patients diagnosed with intrahepatic cholangiocarcinoma at age <45 years have a 39% decreased risk of overall mortality within 5 years from diagnosis compared to patients with cholangiocarcinoma diagnosed at age 45 years or later (HR 0.61 [95% CI 0.54–0.57]).^6^ We observed that young patients were more likely to receive aggressive treatment, including chemotherapy and definitive surgery. This, in addition to their greater reserve, likely explains the improved overall survival among young patients with cholangiocarcinoma.

We found that a greater proportion of young‐onset cholangiocarcinoma patients were non‐White compared with typical‐onset cholangiocarcinoma patients. Demographic shifts have also been described in young‐onset colorectal cancer, which prevalence has risen more rapidly among White compared with Black Americans.^16^ It is possible that certain demographic groups are more susceptible to the effects of known cholangiocarcinoma risk factors, including alcohol and obesity.^17^, ^18^ The complex relationship between gender, race, and carcinogen exposure that may influence demographic shifts in young‐onset cholangiocarcinoma remains to be elucidated.

We found that patients with young‐onset cholangiocarcinoma were more likely to have intrahepatic cholangiocarcinoma, while patients with typical‐onset cholangiocarcinoma were more likely to have extrahepatic cholangiocarcinoma. A relationship between age and tumor location is supported by a US‐based analysis using the SEER database, which found that the mean age of diagnosis for intrahepatic cholangiocarcinoma (67 years) was younger than the mean age of diagnosis of extrahepatic cholangiocarcinoma (72 years).^5^ In contrast, a single‐center analysis of 155 patients with cholangiocarcinoma found that, while intrahepatic cholangiocarcinoma was more common across all age groups, a greater proportion of young patients had extrahepatic cholangiocarcinoma (29%) compared with older patients (17%).^7^ A Japanese analysis characterizing patients under the age of 45 years with sporadic cholangiocarcinoma found that 21% (7 out of 34) had intrahepatic cholangiocarcinoma.^12^ Both these studies were limited by relatively small samples. Kaneko et al. studied a Japanese population, which has distinct differences in cholangiocarcinoma prevalence and risk factors compared with the US population.^16^

Notably, patients with young‐onset cholangiocarcinoma were more likely to be diagnosed with advanced disease. This parallels findings in young‐onset colorectal,^19^, ^20^ gastric,^21^ esophageal,^22^ and pancreatic neuroendocrine^3^ cancers, in which patients under the age of 50 years are consistently more likely to be diagnosed with stage III or IV disease compared with older patients. For example, a recent retrospective cohort study of 269,398 patients with young‐onset colorectal cancer (diagnosed at age 20 to 49 years), found that younger patients were two times more likely (OR 2.04, 95% CI 2.00–2.13) to present with metastatic disease compared with older patients diagnosed at age 70 years or older.^20^ In cholangiocarcinoma, it is unclear whether young‐onset disease represents a more clinically aggressive malignancy or whether cholangiocarcinoma is simply detected later in younger patients. Young‐onset cholangiocarcinoma does appear to have distinct genetic features, including increased frequency of mutations in ASXL1 and KMTC2, compared with typical‐onset cholangiocarcinoma.^7^ Further research on genetic characteristics and the natural history of young‐onset cholangiocarcinoma are needed.

Our study has some notable limitations. We did not have information on several known cholangiocarcinoma risk factors, including primary sclerosing cholangitis, which disproportionately affects younger patients.^23^, ^24^ Information on tobacco exposure, obesity, underlying live disease, performance status, cause of death, and recurrence‐free survival was not available. We did not have information on specific chemotherapeutic treatments and were unable to evaluate treatment of relapsed disease. It is possible that some cases of cholangiocarcinoma were misclassified in this retrospective database analysis. Finally, our data sample was limited to patients who received treatment at a facility participating in the NCDB and thus does not reflect the entire United States population.

To our knowledge, the present study is the largest to date analysis of young‐onset cholangiocarcinoma. Our findings suggest that young‐onset cholangiocarcinoma, similar to other young‐onset gastrointestinal malignancies, is associated with distinct demographic and clinical characteristics. These findings may aid clinicians in identifying young‐onset cholangiocarcinoma. Knowledge of these distinct clinical outcomes in younger populations may aid clinicians in guiding patient expectations and in counseling patients with young‐onset cholangiocarcinoma, a condition for which existing information is limited. Further research on the nature of cholangiocarcinoma in the young, including symptom burden, molecular profile, and efficacy and tolerability of specific treatment regimens, is warranted.

## AUTHOR CONTRIBUTIONS

**Sarah Reddy:** Conceptualization (equal); investigation (equal); methodology (equal); project administration (equal); writing – original draft (equal). **Suleyman Yasin Goksu:** Conceptualization (equal); data curation (equal); formal analysis (equal); investigation (equal); methodology (equal); project administration (equal); resources (equal); software (equal); writing – review and editing (equal). **Nina N. Sanford:** Conceptualization (equal); writing – review and editing (equal). **Radhika Kainthla:** Writing – review and editing (equal). **David Hsiehchen:** Writing – review and editing (equal). **Aravind Sanjeevaiah:** Writing – review and editing (equal). **Amy L. Jones:** Writing – review and editing (equal). **Georgios Karagkounis:** Writing – review and editing (equal). **Salwan Al Mutar:** Writing – review and editing (equal). **Chul Ahn:** Writing – review and editing (equal). **Muhammad S. Beg:** Writing – review and editing (equal). **Syed M. Kazmi:** Conceptualization (equal); investigation (equal); methodology (equal); project administration (equal); supervision (equal); writing – review and editing (equal). Writing assistance: None.

## FUNDING INFORMATION

None.

## CONFLICT OF INTEREST STATEMENT

Reddy, Goksu, Sanford, Kainthla, Hsiechen, Sanjeevaiah, Jones, Karagkounis, Salwan al Mutar, Ahn, Beg: None Reported. Kazmi: Johnson and Johnson.

## Data Availability

The data that support the findings of this study are openly available in the National Cancer Database at https://www.facs.org/quality‐programs/cancer‐programs/national‐cancer‐database/, reference number 8.
